# The ZiBuPiYin recipe regulates proteomic alterations in brain mitochondria-associated ER membranes caused by chronic psychological stress exposure: Implications for cognitive decline in Zucker diabetic fatty rats

**DOI:** 10.18632/aging.103894

**Published:** 2020-11-18

**Authors:** Huiying Xu, Wen Zhou, Libin Zhan, Hua Sui, Lijing Zhang, Chunyan Zhao, Xiaoguang Lu

**Affiliations:** 1 Modern Research Laboratory of Spleen Visceral Manifestations Theory, School of Traditional Chinese Medicine and School of Integrated Chinese and Western Medicine, Nanjing University of Chinese Medicine, Nanjing 210023, China; 2 Institute of Integrative Medicine, Dalian Medical University, Dalian 116044, China; 3 Department of Emergency Medicine, Zhongshan Hospital, Dalian University, Dalian 116001, China

**Keywords:** psychological stress, diabetes-associated cognitive decline, mitochondria-associated ER membrane, proteomics, ZiBuPiYin recipe

## Abstract

Chronic psychological stress (PS) cumulatively affects memory performance through the deleterious effects on hypothalamic-pituitary-adrenal axis regulation. Several functions damaged in cognitive impairment-related diseases are regulated by mitochondria-associated ER membranes (MAMs). To elucidate the role of ZiBuPiYin recipe (ZBPYR) in regulating the MAM proteome to improve PS-induced diabetes-associated cognitive decline (PSD), differentially expressed MAM proteins were identified among Zucker diabetic fatty rats, PSD rats, and PS combined with ZBPYR administration rats via iTRAQ with LC-MS/MS. Proteomic analysis revealed that the expressions of 85 and 33 proteins were altered by PS and ZBPYR treatment, respectively. Among these, 21 proteins were differentially expressed under both PS and ZBPYR treatments, whose functional categories included energy metabolism, lipid and protein metabolism, and synaptic dysfunction. Furthermore, calcium signaling and autophagy-related proteins may play roles in the pathogenesis of PSD and the mechanism of ZBPYR, respectively. Notably, KEGG pathway analysis suggested that ‘Alzheimer's disease’ and ‘oxidative phosphorylation’ pathways may be impaired in PSD pathogenesis, while ZBPYR could play a neuroprotective role through regulating the above pathways. Overall, exposure to chronic PS contributes to the evolution of diabetes-associated cognitive decline and ZBPYR might prevent and treat PSD by regulating the MAM proteome.

## INTRODUCTION

Stress is a necessary, adaptive mechanism for survival in its acute form; however, prolonged stress is considered to be a key issue that impacts health by causing the over-activation of stress-activated systems. Chronic activation of the hypothalamic-pituitary-adrenal (HPA) axis may occur following chronic psychological stress (PS), accompanied by a continuous increase in adrenocorticotropic hormone (ACTH) and glucocorticoid (GC; primarily corticosterone in rodents) concentrations.

Studies have shown that psychological stress is considered as one of the important risk factors of T2DM [1]. From a biological perspective, chronic PS primarily induces insulin resistance by affecting the emotional loop of the hypothalamus and limbic systems. Diabetes-associated cognitive decline (DACD) is generally considered to be a central nervous system (CNS) complication of diabetes, and its pathogenesis primarily includes HPA axis dysregulation, brain insulin resistance, oxidative stress, mitochondrial damage, inflammatory response, and calcium homeostasis imbalance [2, 3]. However, the impact of PS, particularly long-term PS exposure, on DACD has not been thoroughly investigated.

Under a state of chronic PS, the sustained activation of the HPA axis can lead to the simultaneous occurrence of mitochondrial dysfunction and endoplasmic reticulum (ER) stress. Mitochondria and the ER are interrelated both in physiology and function and this link can be attributed to the physical interaction between the two organelles through the mitochondria-associated ER membrane (MAM). MAM is enriched with dozens of proteins and plays an important role in a variety of processes including mitochondrial dynamics and homeostasis, lipid metabolism, calcium homeostasis, and autophagy [4]. Continuing proteomics studies have demonstrated MAM protein changes in mice and humans, as well as in T2DM and cognitive dysfunction [5, 6]. Since MAM functions as a hub for both neurodegeneration [4] and metabolic disease [7], comprehensive knowledge of the protein composition of MAM will be extremely useful in elucidating the mechanisms of PS-induced diabetes-associated cognitive decline (PSD).

The ZiBuPiYin recipe (ZBPYR) is a modification of the Zicheng Decoction, which was recorded in the book of Bujuji written by Cheng Wu in the Qing dynasty, and has been used for the clinical treatment of cognitive impairment. Previous studies from our research group have demonstrated that ZBPYR improves learning and memory in rodents with dementia and DACD, and is related to the regulation of brain insulin resistance, Aβ production and degradation, dendritic spine density, and gut microbiota [8]. Therefore, it was deemed important to explore the molecular mechanisms linking the MAM proteome to PSD and ZBPYR treatment using isobaric tags for relative and absolute quantitation (iTRAQ) with liquid chemistry-mass spectrometry/mass spectrometry (LC-MS/MS) technology.

## RESULTS

### ZBPYR improves glucose metabolism in PSD rats

**Figure 1:**
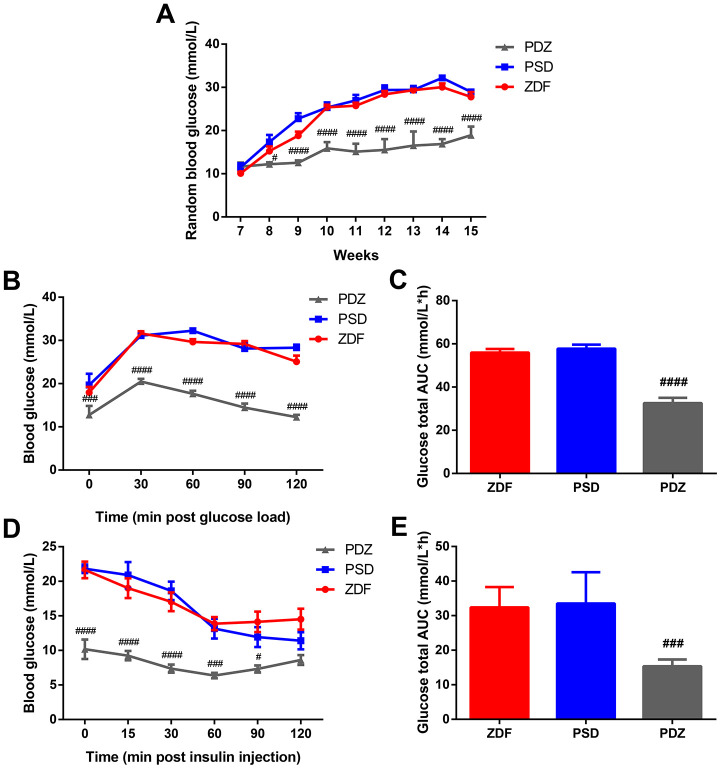
**Effects of ZBPYR on RBG, glucose tolerance, and insulin resistance in PSD rats.** (**A**) RBG was measured at 7-15 weeks old. Oral glucose tolerance tests were performed on 14 hour fasted rats after 10 weeks with a Purina 5008 diet. Blood glucose levels (**B**), total glucose area under the curve (AUC) (**C**). Insulin tolerance tests were conducted on 6 hour fasted animals. Blood glucose levels (**D**), total glucose AUC (**E**). ^#^*p* <0.05, ^###^*p* <0.001, ^####^*p* <0.0001 PDZ vs. PSD. ZDF: ZDF rats; PSD: ZDF rats treated with PS; PDZ: ZDF rats treated with PS combined with ZBPYR administration (mean ± SEM, n=6-8 per group).

Random blood glucose (RBG) levels were not significantly different between ZDF, PSD, and PS combined with ZBPYR administration (PDZ) groups at week seven. Starting at week eight, the levels of RBG were continuously elevated in ZDF and PSD rats, while in the PDZ group they were significantly lower than those in the two other groups (Figure 1A). Within the oral glucose tolerance test (OGTT) and the insulin tolerance test (ITT), there were significant differences in fasting blood glucose levels between the PSD and PDZ groups (*p* <0.001), and the blood glucose in the PDZ group almost returned to the level of 0min at the final time point (Figure 1B–1E). It can be inferred, then, that the administration of ZBPYR obviously reduced blood glucose levels and enhanced insulin sensitivity.

### ZBPYR regulates the activation of the HPA axis in PSD rats

**Figure 2:**
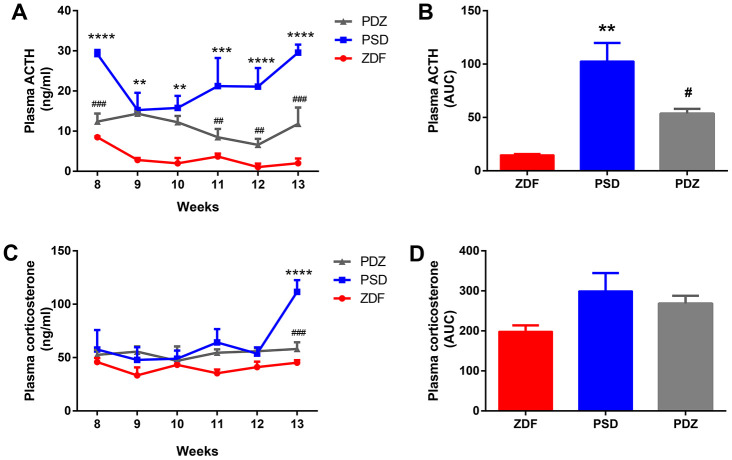
**Effects of ZBPYR on plasma adrenocorticotropic hormone (ACTH) and corticosterone (CORT) during the stress intervention period in PSD rats.** Plasma ACTH values were examined weekly. Plasma ACTH (**A**), ACTH AUC (**B**). Plasma corticosterone values were measured weekly. Plasma corticosterone (**C**), corticosterone AUC (**D**). ^**^p <0.01, ^***^p <0.001, ^****^p <0.0001 PSD vs. ZDF; ^#^*p* <0.05, ^##^*p* <0.01, ^###^*p* <0.001 PDZ vs. PSD (mean ± SEM, n=3 per group).

In weeks 8 to 13, the concentration of ACTH in the PSD group was always significantly higher than that in the ZDF group (*p* <0.01, Figure 2A, 2B). Additionally, the corticosterone (CORT) concentration of the PSD group was significantly higher than that of the ZDF group at week 13 (*p* <0.0001, Figure 2C). Compared with the PSD group, the ACTH concentration in the PDZ group was significantly downregulated at weeks 8, 11, 12, 13, while the CORT concentration was significantly downregulated at week 13 (*p* <0.01, Figure 2A, 2C). Indeed, the administration of ZBPRY significantly attenuated the excessive sustained activation of the HPA axis.

### ZBPYR regulates exploratory behaviors, spatial learning, and memory performance

**Figure 3:**
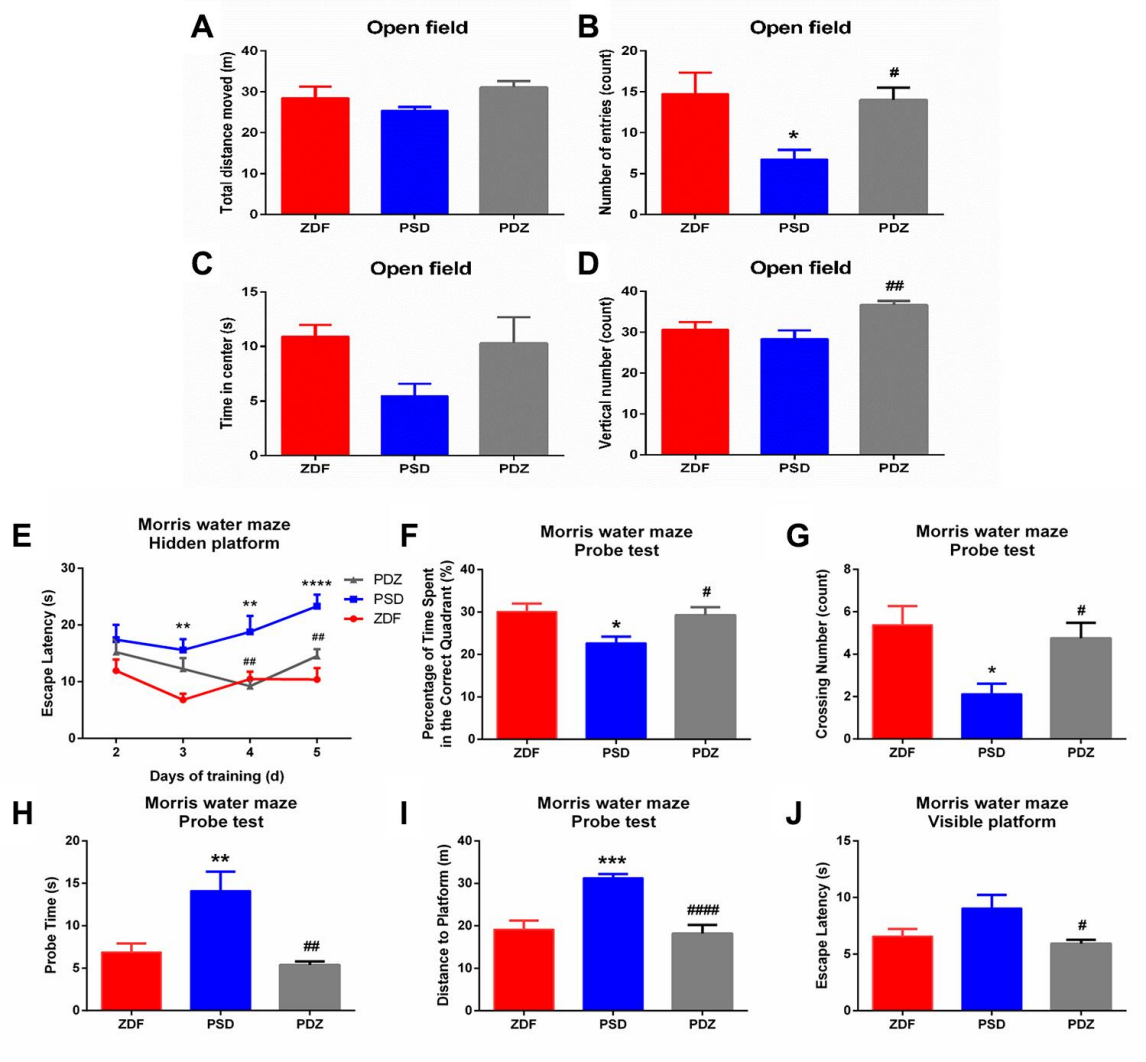
**Effects of ZBPYR on behavioral performance of treatment in PSD rats.** The OFT was performed during day 1 and the MWM was performed during days 2-7 of the last seven days. Spontaneous locomotor activities were recorded in the OFT, including total distance moved (**A**), number of center zone entries (**B**), time spent in the center (**C**), and vertical number (**D**). (**E**) Escape latencies were analyzed in the training trials of the MWM. Performance in the probe test was analyzed, including the percentage of time spent in the target quadrant (**F**), crossing number of the original platform location (**G**), probe time searching for the original platform location (**H**), and the distance traveled to the original platform location (**I**). (**J**) Escape latency in the visible platform version of the MWM was analyzed. ^*^p <0.05, ^**^p <0.01, ^***^p <0.001, ^****^p <0.0001 PSD vs. ZDF; ^#^*p* <0.05, ^##^*p* <0.01, ^####^*p* <0.0001, PDZ vs. PSD (mean ± SEM, n=8 per group).

In the open field test (OFT), the PSD rats entered the center less frequently compared with ZDF rats (*p* <0.05, Figure 3B). ZBPYR treatment significantly increased the number of entries into the center zone and vertical numbers (*p* <0.05, Figure 3B, 3D).

We used the Morris Water Maze (MWM) to measure the effects of different treatments on spatial learning and memory. In the hidden platform test, the escape latency of the ZDF group was shorter than that of the PSD group on days 3-5, while the PSD group escape latency was longer than the PDZ group on days 4 and 5 (*p* <0.01, Figure 3E). In the probe test, compared with the PSD group, the percentage of time spent in the correct quadrant and the platform crossing number were both significantly increased in the PDZ group (*p* <0.05, Figure 3F, 3G). Additionally, PDZ rats swam shorter distances and required less time to locate the original platform position (*p* <0.01, Figure 3H, 3I). In the visible platform version of the test, the escape latency of the PDZ group was shorter than that of the PSD group (*p* <0.05, Figure 3J). Indeed, ZBPYR administration evidently improved spontaneous locomotor activities and cognitive decline in the PSD group.

### Validation of the purity of MAM fractions

**Figure 4:**
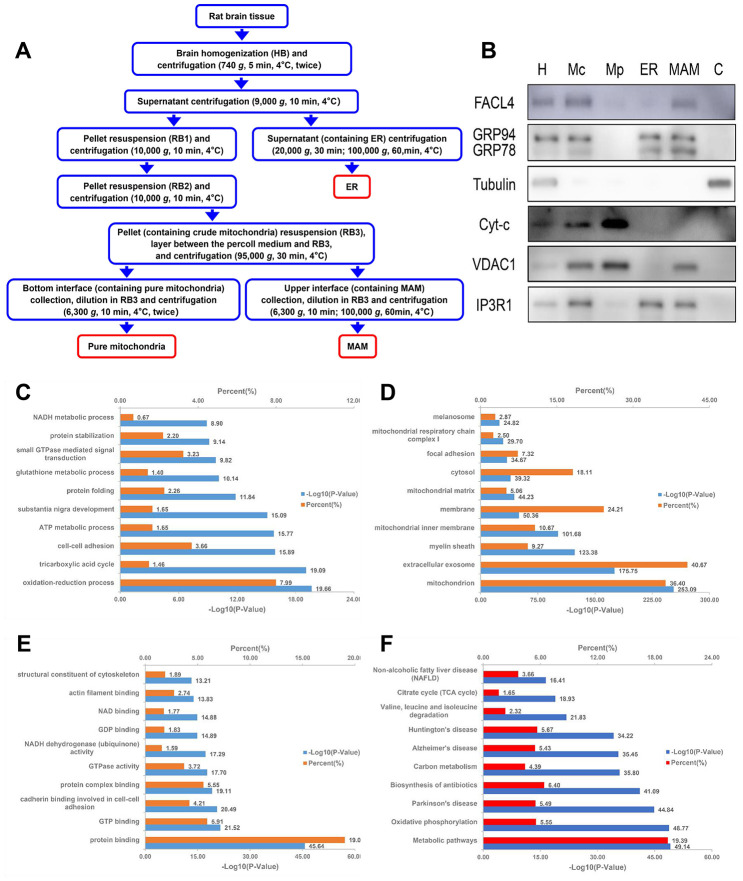
**Schematic diagram of MAM isolation and purity confirmation of the MAM fraction by WB and enrichment analysis.** (**A**) MAM was isolated from ZDF rat brains by applying differential centrifugation and self-forming Percoll gradient centrifugation. Other cell organelles, such as crude mitochondria, pure mitochondria, and ER were similarly obtained following the multiple centrifuge steps. (**B**) Western blot analysis of organelle markers in isolated MAM from the brain were enriched for FACL4 and KDEL, and were free from tubulin and cytochrome-C contamination. H: homogenate; Mp: pure mitochondria; Mc: crude mitochondria; ER: endoplasmic reticulum; MAM: mitochondria-associated ER membrane; C: cytosol. DAVID Gene Ontology enrichment analysis of MS-identified proteins. The most enriched (top 10) biological pathways (**C**), cellular components (**D**), and molecular functions (**E**) of the ranked protein list. (**F**) KEGG pathway enrichment analysis of MS-identified proteins.

To validate the quality of MAM preparations from rat brains, three independent methods were used. First, the different steps of MAM preparation from ZDF rat brains (n=3) were quality controlled by western blot analysis (Figure 4A, 4B). The distribution trend of seven molecules, such as the MAM marker acyl-CoA synthetase long chain 4 (ACSL4/FACL4), in different fractions was consistent with previously published data [9, 10]. Secondly, LC-MS/MS protein identification of MAM preparations revealed that MAM fractions contained five known characteristic MAM proteins, namely Apoe, Fis1, S100B, Cisd2, and Bcap31 (Supplementary Table 1). Furthermore, 32 of these proteins are associated with cognitive impairment and 12 are associated with diabetes mellitus. Finally, according to the enrichment analysis of MAM samples, most of the enriched cellular components (e.g., mitochondrion and the ER), biological processes (e.g., cell redox homeostasis and glucose metabolic processes), molecular functions (e.g., GTP binding, NAD binding, and GTPase activity), and Kyoto Encyclopedia of Genes and Genomes (KEGG) pathways (e.g., Alzheimer's disease and oxidative phosphorylation) are strongly related to mitochondrial-ER processes (Figure 4C–4F).

### Differentially-expressed brain MAM proteins between PSD:ZDF and PDZ:PSD groups

**Figure 5:**
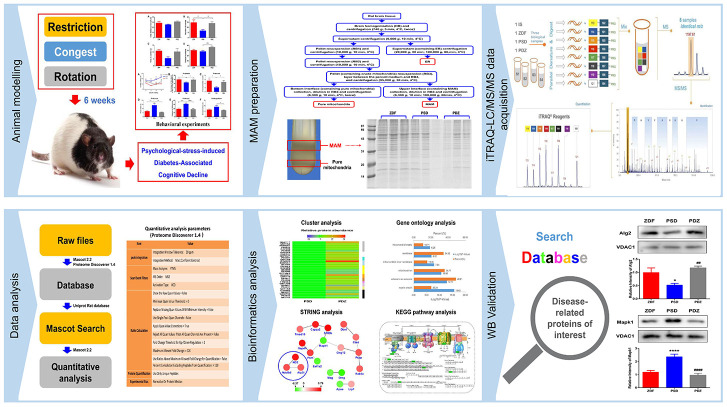
**Proteomics workflow.** An animal model of PS-induced diabetes-associated cognitive decline was established by exposure to three chronic psychological stressors. Fresh brain tissue MAM was extracted using gradient centrifugation, sample lysis was performed, and proteins were detected by an iTRAQ 8-plex. Following digestion and labelling, the samples were pooled, the peptides were fractionated by EASY-nLC1000 chromatography, and fractions were subsequently analyzed by standard LC-MS/MS. Peptides were identified and quantified based on their iTRAQ reporter area and relative protein quantification was inferred from these values. Key proteins were screened based on bioinformatics analysis and public databases were searched to further screen for differentially expressed proteins closely related to disease, for validation.

**Figure 6:**
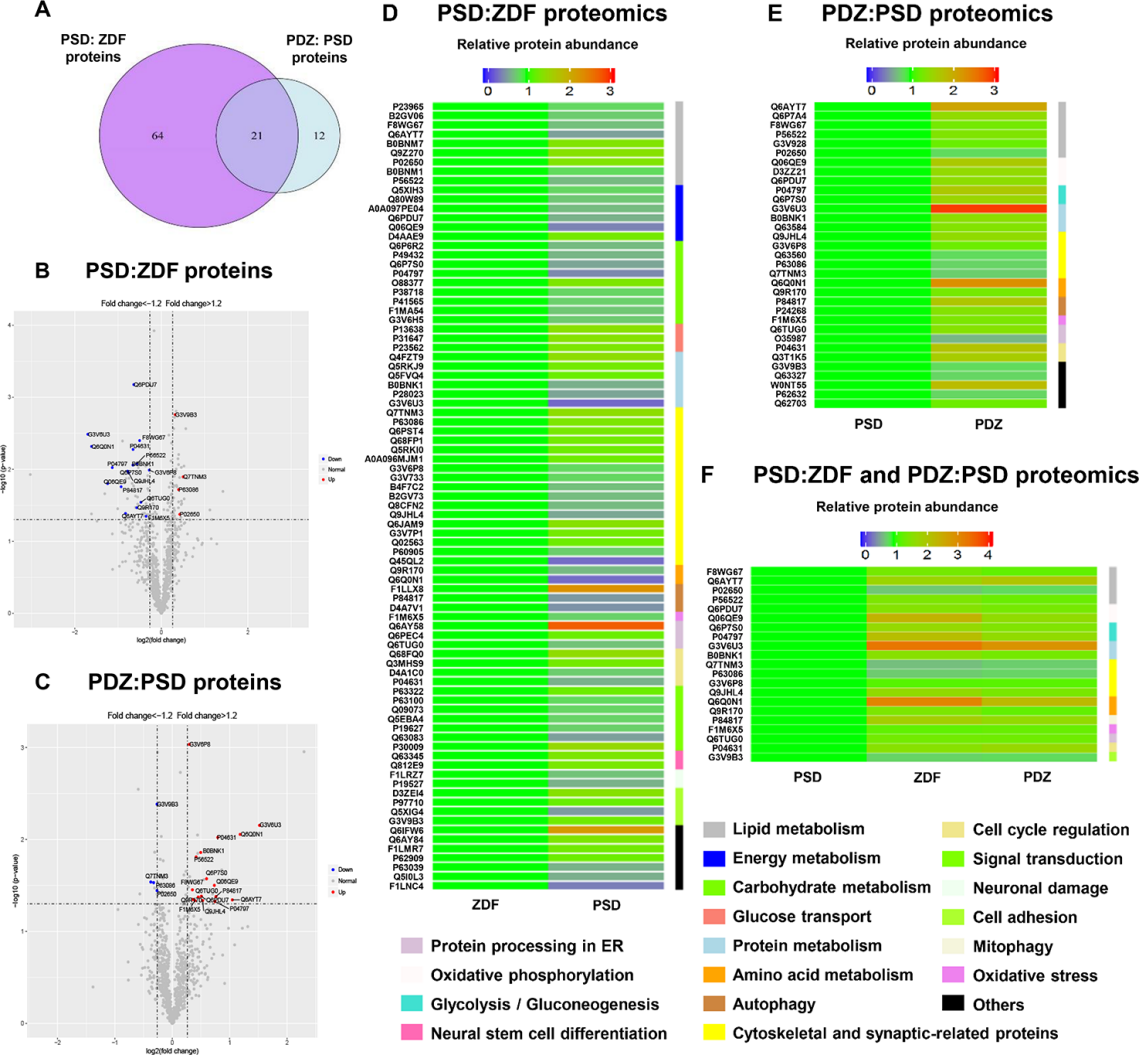
**Analysis and comparison of the brain MAM proteins between PSD:ZDF and PDZ:PSD groups.** (**A**) Using a fold change cutoff of ≥1.2 and a p-value cutoff of ≤0.05, a Venn diagram was constructed to find differentially regulated proteins in the PSD:ZDF, PDZ:PSD, or both. Volcano plots showing log2 fold-change (x-axis) and -log10 p-value (y-axis) were used for all quantified proteins in the PSD:ZDF (**B**) or PDZ:PSD group (**C**). Colored dots indicate proteins commonly expressed in both groups and the upregulated and downregulated proteins are labeled by red and blue, respectively. (**D**, **E**) Chronic PS caused 85 differentially expressed proteins and ZBPYR treatment resulted in 33 proteins to be differentially expressed. (**F**) Twenty-one overlapped proteins were differentially expressed in both groups (n=3 per group).

The entire proteomics workflow is shown in Figure 5. A total of 1,819 proteins were identified and 938 common proteins were successfully quantified from all individual samples. Both Venn diagram and Volcano plots both showed that chronic PS and ZBPYR treatment caused differential expressions of 85 and 33 proteins in brain MAMs, respectively. Furthermore, twenty-one overlapped proteins were differentially expressed in both PSD:ZDF and PDZ:PSD groups (Figure 6A–6C). Among the 85 proteins differentially expressed in the PSD:ZDF group, 36 of those were increased and 49 decreased (Figure 6D, Supplementary Table 2). The significantly increased MAM proteins included Bcap31, Lamp2, Krt10, Marcks, and Cct5, while those significantly decreased included Alg2, Gnb4, Cndp2, Vti1b, and ND2. Moreover, among the 33 proteins differentially expressed in PDZ:PSD group, 25 of those were increased and eight decreased (Figure 6E, Supplementary Table 3). The significantly increased MAM proteins included Alg2, Cndp2, Abhd12, Slc4a10, and S100b, while those significantly decreased included Nsfl1c, OMG, Mapk1, Mobp, and Eef1a2. Additionally, there were 21 overlapped proteins, namely Acot7, Abhd12, Apoe, Fdxr, Atp5, ND2, Pkm, Gapdh, Alg2, Rab5c, OMG, Mapk1, Gng12, Dbnl, Cndp2, Bcat1, Fis1, Txnrd2, Dnajb11, S100b, and Mag (Figure 6F, Supplementary Table 4).

### Bioinformatics analysis of differentially expressed brain MAM proteins

Deep analyses based on the differentially expressed proteins (DEPs) including a gene ontology (GO) enrichment analysis, PPI network analysis, and KEGG pathway analysis, were also conducted.

**Figure 7:**
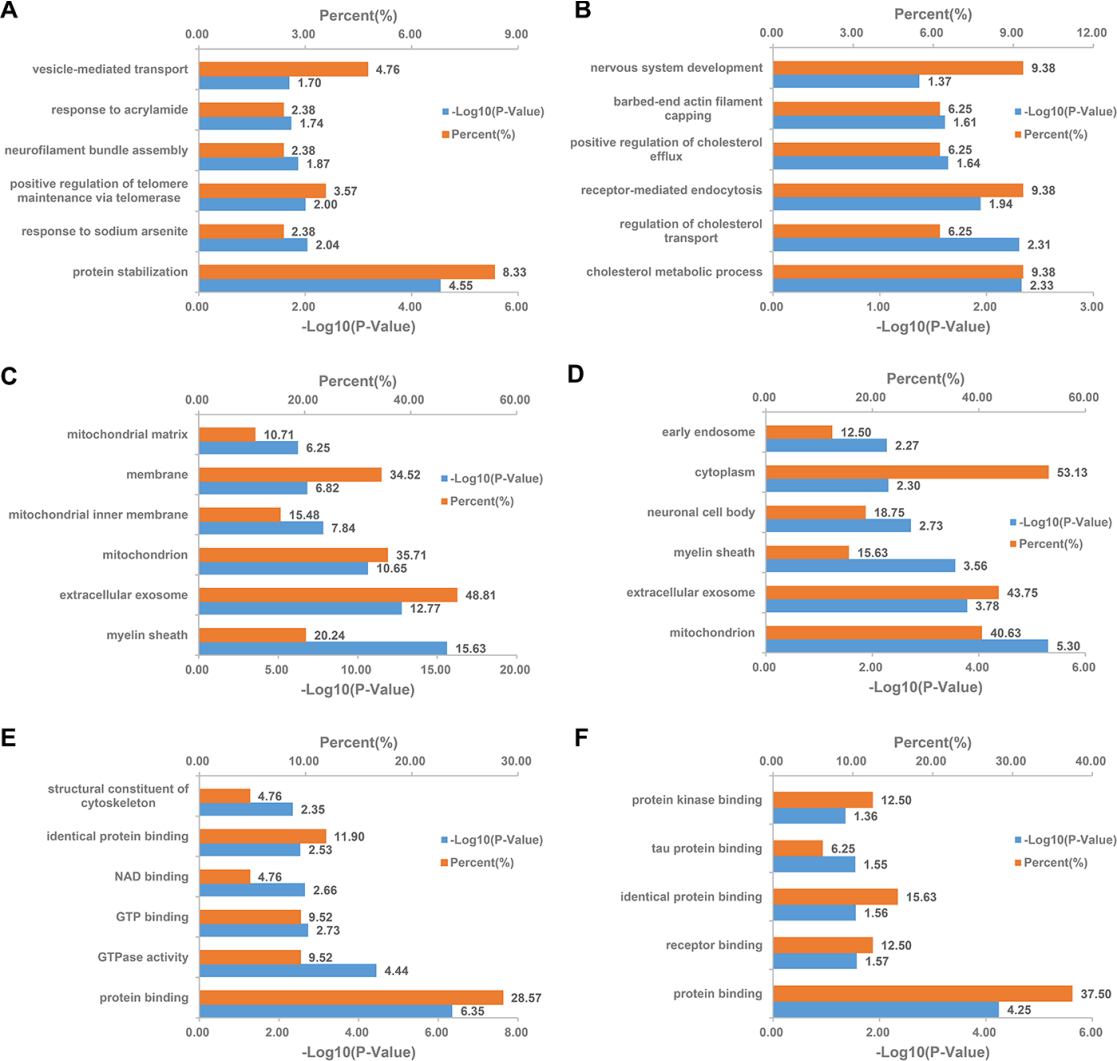
**DAVID Gene Ontology enrichment analysis for the differentially expressed MAM proteins in PSD and PDZ rats.** Enrichment analysis for MAM proteomics in the PSD:ZDF group by biological processes (**A**), cellular component (**C**), and molecular function (**E**), respectively. Enrichment analysis for MAM proteomics in the PDZ:PSD group by biological processes (**B**), cellular component (**D**), and molecular function (**F**), respectively.

In PSD:ZDF rats, altered biological processes included the strong enrichment of protein stabilization, response to sodium arsenite, and positive regulation of telomere maintenance via telomerase (Figure 7A). Cellular component annotation demonstrated a strong enrichment of the myelin sheath, extracellular exosome, and mitochondrion (Figure 7C). An analysis based on the molecular functions showed that the majority of the DEPs were associated with two major functions. One of these functions is binding, including protein binding, GTP binding, NAD binding, and identical protein binding. The other function is catalyst activity, including GTPase activity (Figure 7E). In the PDZ:PSD group, the biological process revealed that the DEPs primarily involved in cholesterol homeostasis, including cholesterol metabolic processes, regulation of cholesterol transport, and positive regulation of cholesterol efflux (Figure 7B). Additionally, a GO analysis showed similar results with those in the PSD:ZDF group in regards to cellular components and molecular functions (Figures 7D, 7F).

**Figure 8:**
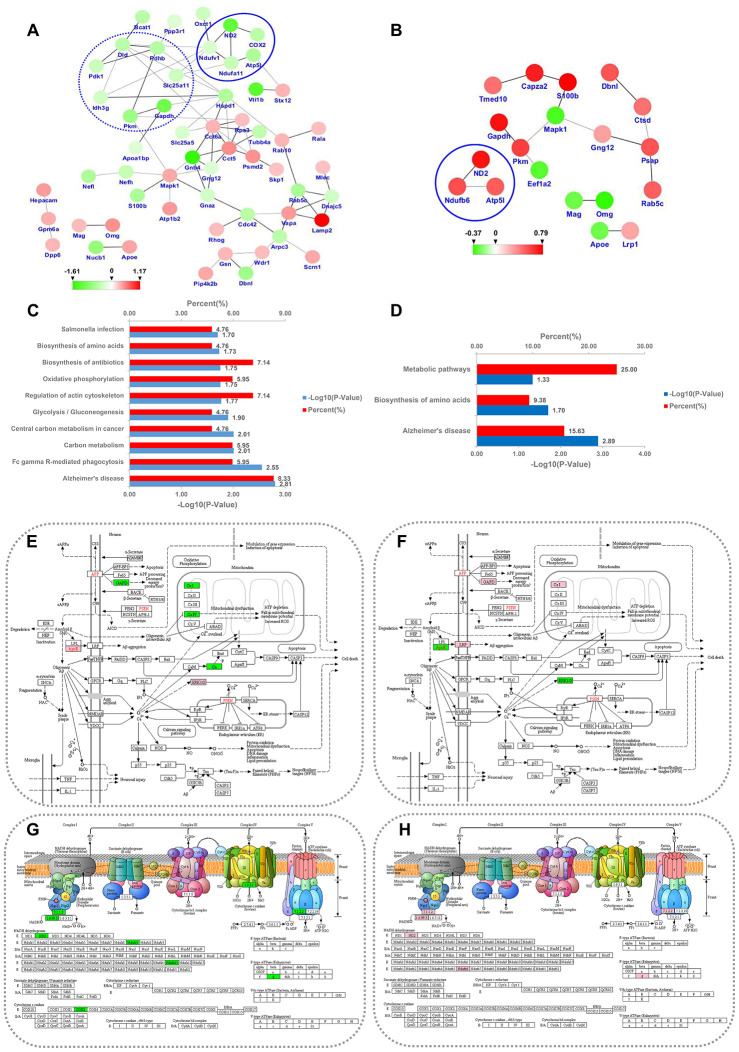
**PPI network and KEGG pathway analysis for the differentially expressed MAM proteins in PSD and PDZ rats.** Intricate PPI networks among the differential proteins in the PSD:ZDF (**A**) and PDZ:PSD (**B**) groups. Balls represent proteins, some of which are increased (red balls), others decreased (green balls). KEGG pathway enrichment analysis for differential proteins in the PSD:ZDF (**C**) and PDZ:PSD (**D**) rats. Alzheimer's disease pathway map in PSD:ZDF (**E**) and PDZ:PSD (**F**) rats, respectively. Oxidative phosphorylation pathway map in PSD:ZDF (**G**) and PDZ:PSD (**H**) rats, respectively. The upregulated and downregulated proteins are labeled in pink and green, respectively.

A PPI network analysis showed that interactions between proteins related to carbohydrate metabolism were evident (e.g., Dld, Pdhb, Pkm, Gapdh, Idh3g, Pdk1, and Slc25a11) in the PSD:ZDF group. Additionally, interactions among oxidative phosphorylation-related proteins were found both in the PSD:ZDF (i.e., Ndufv1, Ndufa11, COX2, Atp5l, and ND2) and the PDZ:PSD (i.e., ND2, Ndufb6, and Atp5l) groups (Figure 8A, 8B).

The DEPs were classified into 22 pathways in the PSD:ZDF group and five pathways in the PDZ:PSD groups. In the PSD:ZDF group, KEGG pathway annotation revealed a strong enrichment of pathways such as Alzheimer's disease (AD) and oxidative phosphorylation (OXPHOS; Figure 8C). There were seven DEPs (i.e., Apoe, Ndufv1, Ndufa11, COX2, Gapdh, Mapk1, and Ppp3r1) observed in the AD pathway and five DEPs (i.e., Ndufv1, Ndufa11, COX2, Atp5l, and ND2) were observed in the OXPHOS pathway (Figure 8E, 8G). In the PDZ:PSD group, the AD pathway was also strongly enriched (Figure 3D). Furthermore, five DEPs (i.e., Lrp1, Apoe, Ndufb6, Gapdh, and Mapk1) were found in the AD pathway and three proteins (i.e., ND2, Ndufb6, and Atp5l) were found in the OXPHOS (Figure 8F, 8H).

### Representative proteins differentially expressed in brain MAM

**Figure 9:**
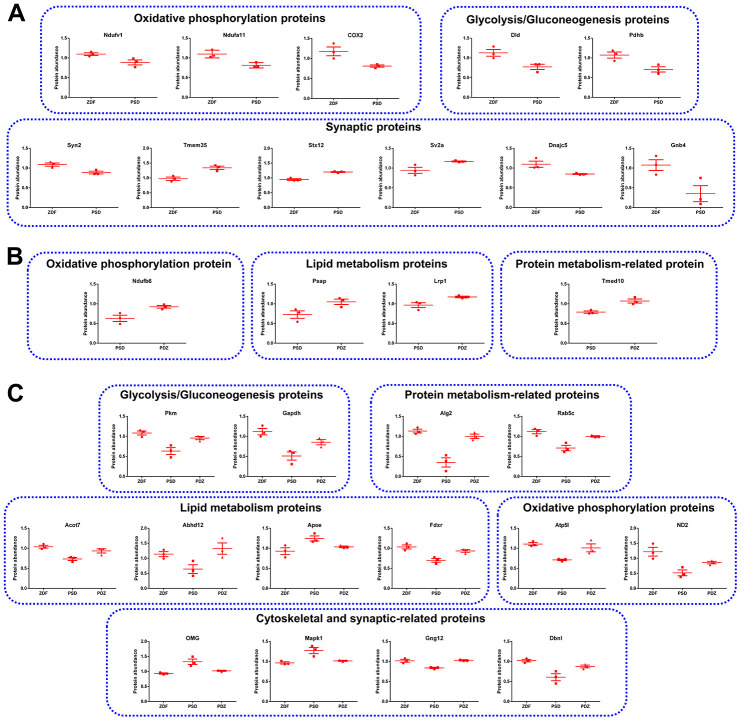
**Representative MAM proteins that were differentially expressed in PSD and PDZ rats.** (**A**) Proteins that were abnormally expressed in PSD rats. (**B**) Proteins that were differentially expressed in PDZ rats. (**C**) MAM proteins that were differentially expressed in both PSD and PDZ rats (mean ± SEM, n=3 per group).

Based on the results of functional analysis, 11, 4, and 14 representative proteins in the PSD:ZDF, PDZ:PSD and PSD:ZDF/PDZ:PSD combined groups were selected for protein abundance analysis, respectively (Figure 9). The common proteins in both groups are the glycolysis/gluconeogenesis proteins Pkm and Gapdh, protein metabolism-related proteins Alg2 and Rab5c, lipid metabolism proteins Acot7, Abhd12, Apoe, and Fdxr, OXPHOS proteins Atp5l and ND2, and cytoskeletal and synaptic-related proteins OMG, Mapk1, Gng12, and Dbnl (Figure 9C).

### Validation of selected DEPs by western blotting

**Figure 10:**
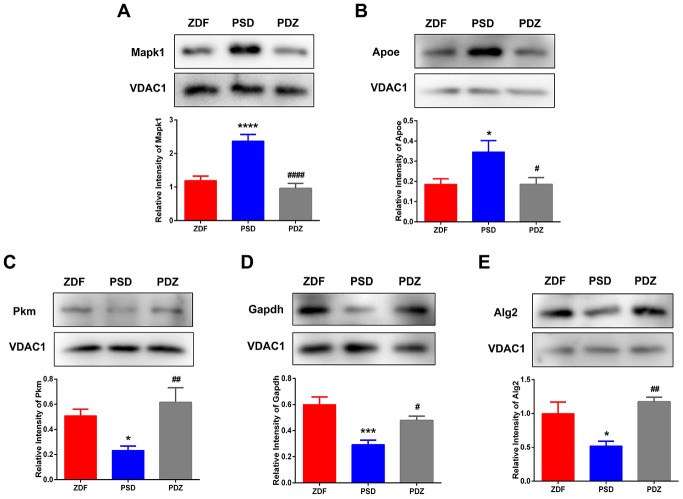
**Western blotting analysis for selected differentially expressed proteins.** MAM fractions from ZDF, PSD, and PDZ rats were analyzed by western blotting using antibodies against mitogen-activated protein kinase 1 (Mapk1) (**A**), apolipoprotein E (Apoe) (**B**), pyruvate kinase (Pkm) (**C**), glyceraldehyde-3-phosphate dehydrogenase (Gapdh) (**D**), and ALG2, alpha-1,3/1,6-mannosyltransferase (Alg2) (**E**). VDAC1 was used to ensure equal protein loading and transfer. The levels of Pkm, Mapk1, Apoe, Gapdh, and Alg2 were normalized relative to VDAC1 levels. ^*^p <0.05, ^***^p <0.001, ^****^p <0.0001 PSD vs. ZDF; ^#^*p* <0.05, ^##^*p* <0.01, ^####^*p* <0.0001 (mean ± SEM, n=3 per group).

To verify the quantitative results of the iTRAQ-LC/MS/MS experiments, western blotting experiments were performed on five selected DEPs (Apoe, Mapk1, Pkm, Gapdh, and Alg2). There were significantly increased levels of Apoe and Mapk1 in PSD:ZDF rats (Figure 10A, 10B) and significantly decreased levels in PDZ:PSD rats. These experiments also demonstrated that Pkm, Gapdh, and Alg2 expression was decreased sharply in the PSD:ZDF group and that ZBPYR treatment enhanced their expression (Figure 10C–10E).

## DISCUSSION

Chronic PS as a common factor in modern life increases the risk of T2DM, but its pathogenesis in T2DM and DACD, generally considered to be a CNS complication of diabetes, is still unclear. In current study, a proteomic analysis on brain MAM samples was adopted to identify protein expression differences in animals with PSD and also to assess the effects of ZBPYR on PSD. We used male Zuker diabetes fatty (ZDF) rat as T2DM model, which is initiated with a mutation in the leptin receptor gene. When fed with a diet of Purina 5008, the ZDF rats exhibit the following characteristics: hyperglycemia, which develops between 7 and 10 weeks of age; early hyperinsulinemia, which rapidly falls as the beta cells fail; Insulin resistance and abnormal glucose tolerance, which becomes progressively worse with age; and spatial cognitive impairment, which occurs at 16 weeks of age.

### Chronic PS aggravates diabetes and cognitive impairment

High concentrations of GCs enhance the risk of developing T2DM by increasing hepatic glucose production and reducing insulin secretion and sensitivity [11], while also increasing the risk of cognitive impairment through hippocampal neuronal loss, decreased neurogenesis, and dendritic atrophy [12]. In the current study, PSD rats demonstrated an increasing trend in blood glucose levels, sustained activation of the HPA axis, and both behavioral and cognitive anomalies. These changes were associated with 85 MAM proteins that were significantly modulated in the brains of PSD rats. Bioinformatics analyses demonstrated that the modulated proteins were involved in OXPHOS, glycolysis/gluconeogenesis, synaptic function, protein metabolism, lipid metabolism, cytoskeleton, glucose transport, and calcium signaling. Additionally, the current study suggests that elevations in CORT and ACTH occur prior to the development of glucose intolerance and may facilitate the succeeding onset of hyperglycemia in ZDF rats. Average blood glucose levels in PSD rats reached diabetes levels (blood glucose ≥ 16.7 mmol/L) at eight weeks of age, while ZDF rats did not meet this level (PSD: 17.34 ± 1.63 vs. ZDF: 15.26 ± 1.04 mmol/L). At nine weeks of age, the mean blood glucose levels in both groups exceeded 16.7 mmol/L (PSD: 22.76 ± 1.26; ZDF: 18.81 ± 0.94 mmol/L).

### ZBPYR treatment alleviates diabetes and cognitive impairment

A previous study conducted by our research group showed that the benefit of ZBPYR on DACD may be achieved through improvements in dendritic spine density, reduction of Aβ_1-42_ deposition, attenuation of brain leptin and insulin signaling pathway injury, and maintenance of gut microbiota homeostasis. In the current study, ZBPYR significantly improved the chronic activation of the HPA axis, reduced blood glucose concentrations, promoted insulin sensitivity, slowed the progression of diabetes, as well as enhanced exploratory behavior, learning, and memory performance in PSD rats. These changes were associated with 33 MAM proteins that were significantly modulated in the brains of PDZ rats. Bioinformatics analyses showed that the modulated proteins were involved in OXPHOS, glycolysis/gluconeogenesis, synaptic function, protein metabolism, lipid metabolism, and protein processing in the ER.

### Chronic PS and ZBPYR treatment result in the common differential expression of brain MAM proteins in ZDF and PSD rats

### Energy metabolism

Chronic PS is associated with abnormal energy metabolism [13]. In the current study, four proteins associated with energy metabolism, namely Gapdh, Pkm, Atp5l, and ND2 were downregulated in the PSD group. The inhibition of Gapdh activity can contribute to apoptosis, a common phenomenon in cognitive impairment, including in AD, through the glycolytic generation of toxic side-products [14]. PKM plays a prominent role in the maintenance of different forms of persistent, long-term synaptic plasticity [15] and is used as a biomarker for detecting disease progression and therapeutic response in AD [16]. ATP5L is downregulated in the choroid plexus in those with AD, while reduced choroid plexus function impaired the clearance of toxic metabolites and may facilitate neuronal death during AD progression [17]. ND2 is the core component of mitochondrial respiratory chain complex I. Previous studies have shown that mRNA levels of mitochondrial genome-encoded COX I is significantly decreased in the temporal cortex in those with AD [18]. The levels of these four proteins were upregulated in the PDZ group, suggesting that ZBPYR improved PSD, potentially by regulating energy metabolism, including glycolysis and OXPHOS.

### Lipid metabolism

Chronic PS has been shown to affect lipid metabolism [19]. In the current study, chronic PS caused a simultaneous decrease of Acot7 and Abhd12 in the PSD group, but increased Apoe. Apoe plays an important role in lipid transport in the central nervous system, regulating neuronal survival and sprouting [20]. Genome-wide association studies have confirmed that APOE gene polymorphisms are closely linked to the risk of AD and the APOE ε4 allele is the strongest genetic risk factor for AD [21]. Acot7 is highly expressed in neurons, counter-regulates fatty acid metabolism in neurons, and protects against neurotoxicity [22]. In line with this, Acot7N^-/-^ mice exhibit neurological dysfunction and neurodegeneration. Abhd12 is an enzyme that catalyzes the hydrolysis of 2-arachidonoyl glycerol (2-AG). Furthermore, 2-AG regulates neurotransmission and neuroinflammation [23]. The pathological processes of many diseases are connected with inflammation, including DM and cognitive impairment. Given that expression changes of three proteins in the PSD group was the inverse of that in the PDZ group, alternated protein expression affecting lipid metabolism is plausibly a common phenomenon observed in animals with cognitive dysfunction.

### Protein metabolism

Chronic PS has been demonstrated to affect protein metabolism [24]. Changes in protein metabolism are essential to disease onset and progression in many neurodegenerative diseases, such as AD [25]. In the current study, the levels of Alg2 and Rab5c were decreased in the PSD group, while their levels were elevated in the PDZ group. Alg2 acts as an alpha-1,3 mannosyltransferase. Defects in this gene have been associated with the congenital disorder of glycosylation type Ih, which results in a wide variety of clinical features such as defects in nervous system development. Rab5c is a small GTPase of the Ras superfamily. Higher Aβ production has also been observed in Rab5-endocytic vesicles in the early endosome and lysosomes in the brain of those with AD, whereas silencing of only the Rab5C isoform significantly increases APP, soluble APP, and Aβ_42_ levels [26]. The current results suggest that ZBPYR improved PSD potentially by regulating protein metabolism, including post-translational modifications.

### Synaptic dysfunction

A high level of glucocorticoids, induced by chronic stress, causes specific synaptic deficits in the hippocampus, accompanied by stress-induced behavioral dysfunction [27]. In the current study, chronic PS indeed caused synaptic dysfunction with increased levels of Mapk1, OMG, and Gng12 and decreased Dbnl levels, while ZPBYR treatment stabilized their expressions. MAPK1 plays a key role in cellular proliferation and differentiation during neural development, as well as in cognition and memory formation [28]. Moreover, the expression of MAPK1 is significantly upregulated in the brains of AD patients and in rodent models [29], leading to synaptic plasticity damage and impairment of learning and memory capacity. OMG is a myelin-associated inhibitor (MAI). MAIs stabilize synaptic ultrastructures by modulating cytoskeletal rearrangements and suppressing activity- and experience-dependent synaptic plasticity [30]. Furthermore, increased MAI expression is implicated in a number of neurological conditions, including AD. Gng12 is known as the γ12 subunit of G proteins. Results from one functional gene group approach suggest that the involvement of synaptic heterotrimeric G proteins in cognitive ability and alterations in synaptic signaling processes could explain this correlation [31]. Taken together, these findings suggest that ZPBYR treatment improves PSD plausibly by regulating the synaptic dysfunction associated with the alternated protein expression of Mapk1, OMG, Gng12, and Dbnl.

### Chronic PS leads to specific changes in brain MAM proteins in ZDF rats

### Calcium signaling

Voltage gated Ca^2+^ channels are among the most pronounced targets of corticosteroid hormones. Deregulation of intracellular calcium signaling has been implicated in the pathogenesis of AD. In the current study, chronic PS caused a simultaneous decrease in Ppp3r1, Slc25a5, Nipsnap1, and Nucb1 in the PSD group. Ppp3r1 encodes a regulatory subunit of calcineurin. In animal models, the disruption of calcineurin activity has severe effects on memory [32]. Furthermore, a genetic variant of Ppp3r1 is strongly associated with the rapid progression of AD in humans [33]. Slc25a5 and Nipsnap1 play prominent roles in signal transduction and postsynaptic density function regulation [34, 35], therefore, the abnormal expression of these genes can result in memory impairment. Nucb1 inhibits the aggregation of islet-amyloid polypeptide associated with T2DM, and Aβ_42_ associated with AD by stabilizing their respective protofibril intermediates [36]. In post-mortem brains of AD patients, NUCB1 protein levels have been found to be reduced by an average of 50% compared to controls. The current results demonstrate that decreased protein expression of the calcium signaling pathway could plausibly contribute to DACD.

### ZBPYR treatment causes specific changes of brain MAM proteins in PSD rats

### Autophagy

The current results also showed that an autophagy-related protein, Ctsd, was upregulated in the PDZ group. Ctsd, a lysosomal protease, is involved in the degradation of the APP protein, the processing of Aβ peptides and tau, and the clearance of amyloid plaques *in vitro* [37–39]. Consistent with previous results, our research group has previously shown that ZBPYR treatment improves Spleen-yin deficiency DACD by promoting the occurrence of autophagy [40]. Therefore, we have reason to believe that ZBPYR improved DACD potentially by influencing autophagy, and that Ctsd may be a molecular target of the neuroprotective effects of ZBPYR.

### Regulation of signaling pathways enriched in DEP datasets

### Alzheimer's disease (AD) pathway

The current data suggested that two upregulated DEPs (Apoe and Mapk1) and five downregulated DEPs (Ndufv1, Ndufa11, COX2, Gapdh, and Ppp3r1) were involved in Alzheimer's disease signaling in the PSD:ZDF group, while three upregulated DEPs (Lrp1, Ndufb6, and Gapdh) and two downregulated DEPs (Apoe and Mapk1) were observed in the PDZ:PSD group. Alzheimer's disease pathway maps showed that chronic PS may result in Aβ aggregation, mitochondrial dysfunction, decreased energy production, and apoptosis, while ZBPYR administration could improve above dysfunction. Alzheimer’s disease (AD) is a chronic disorder that slowly destroys neurons and causes serious cognitive disability. It is also associated with senile plaques and neurofibrillary tangles. Similarly, clinical studies have shown that amyloid plaques and neurofibrillary tangles are present in the brain tissue of patients with T2DM and the density of amyloid plaques is closely related to the course of T2DM [41]. Consistent with this, animal experiments have confirmed that diabetic mice not only have learning and memory dysfunction, but that their brain neurons have AD-specific pathological changes [42]. Our proteomics data is partially consistent with this mechanism, suggesting that ZPBYR treatment improved PSD potentially by regulating the abnormal protein expression of the Alzheimer’s disease pathway.

### Oxidative phosphorylation (OXPHOS) pathway

We also found that five downregulated DEPs (Ndufv1, Ndufa11, COX2, Atp5l, and ND2) and three upregulated DEPs (ND2, Ndufb6, and Atp5l) were involved in Oxidative phosphorylation signaling in the PSD:ZDF and PDZ:PSD groups, respectively. Given that expression change in the PSD:ZDF group was opposite of that in the PDZ:PSD group, it follows that chronic PS and ZBPYR administration may interfere with the coordination of energy protein mechanism (gene/protein expression) in MAM among the two groups. Oxidative phosphorylation is a way for mitochondria to supply energy metabolism when sufficient oxygen is provided. Mitochondrial dysfunction primarily manifests as disordered energy metabolism, perturbations in the electron transport chain, synaptic dysfunction, and neuronal apoptosis. Energy metabolism disorder has been demonstrated in brain injury caused by diabetes. Clinical trials and animal studies have shown that the activity of mitochondrial complex I-IV in the brain of those with AD decreased when accompanied with abnormal oxygen consumption [43, 44]. Additionally, a previous proteomic study from our research group demonstrated that several molecules closely related to energy metabolism changed in the hippocampus of the DACD model rats, suggesting a possible energy metabolism disorder in the model rat [45]. Collectively, this evidence points to the notion that perturbations of mitochondrial energy metabolism-related proteins responsible for ATP generation via oxidation phosphorylation play an important, perhaps crucial, role in the development of DACD and treatment of ZBPYR.

### Study advantages and limitations

Compared with previous studies, the merits of this work could be summarized by four characteristics, including the closer animal model to the pathogenesis of DACD in humans, the more sophisticated proteomic technique, the more precise subjects—MAM fractions from brain, and stress stimulation with reduced potential adaptability. First, ZDF rat has been used as a classic model to study the pathogenesis of T2DM caused by obesity and insulin resistance and it has been shown to have spatial cognitive impairment by 16 weeks of age. In other words, the ZDF rat is a promising animal model by which to study DACD. Furthermore, almost all of previous studies have focused on the MAM profiles of the mouse with normal physiological and disease states [5, 6, 46], while there are currently no studies focused on the effects of psychological stress and drug administration on the rat MAM proteome. Therefore, we chose ZDF rats as the research objects. Second, a number of previous studies have analyzed proteins in the MAM fraction using different proteomics techniques, such as one-dimensional gel electrophoresis, label-free, and iTRAQ-labeled technology combined with LC-MS/MS [5, 47, 48]. Based on the above background, we selected the more sophisticated and sensitive proteomics approach, namely iTRAQ-based LC-MS/MS, to reveal protein changes. Third, many proteomics studies have been performed at the levels of the whole cell or tissue lysates. It may be better to understand the essential regulation mechanisms and biological functions of MAM with a spatio-temporal resolution through subcellular fractionation. It is of importance to note that since MAM is a hub for both neurodegeneration and metabolic disease, it is useful to select brain MAM for further proteomics research to understand the pathogenesis of PSD and the mechanism of ZPBYR. Finally, chronic PS exposure is defined as a prolonged period of stress, during which an animal is exposed to a continuous or repeated psychological stressor without habituation. Habituation, however, can be very hard to predict given that it depends on the interval between stressors as well as the intensity, duration, predictability, and types of stressors used. Therefore, by observing physical status and indicators of rats, we continuously adjusted the interval and duration of stress intervention to minimize the possibility of adaptation or habituation.

However, there are also limitations to the chosen study design. First, although animal models are essential to preclinical trials, substantial work is needed in preclinical and human studies to fully illustrate the effects of PS and ZBPYR on patients with T2DM and DACD. Additionally, due to the particularity of the strain, the current study was restricted to male ZDF rats; however, we cannot exclude that the protein profiles may differ in female animals. Indeed, studies have shown that gender differences in neuropsychological endocrine activation make females more prone to the effects of long-term psychosocial stress on health [49]. Furthermore, although the current study has found common and specific differential proteins caused by PS and ZBPYR, the exact mechanisms by which this occurred could not be directly determined. In particular, there was a lack of evidence from animals with specific gene knockouts to determine whether the effects of the two interventions on lipid and protein metabolism, energy metabolism, synaptic function, autophagy, and calcium signaling pathways were direct effects or in association with a signaling cascade.

## CONCLUSIONS

**Figure 11:**
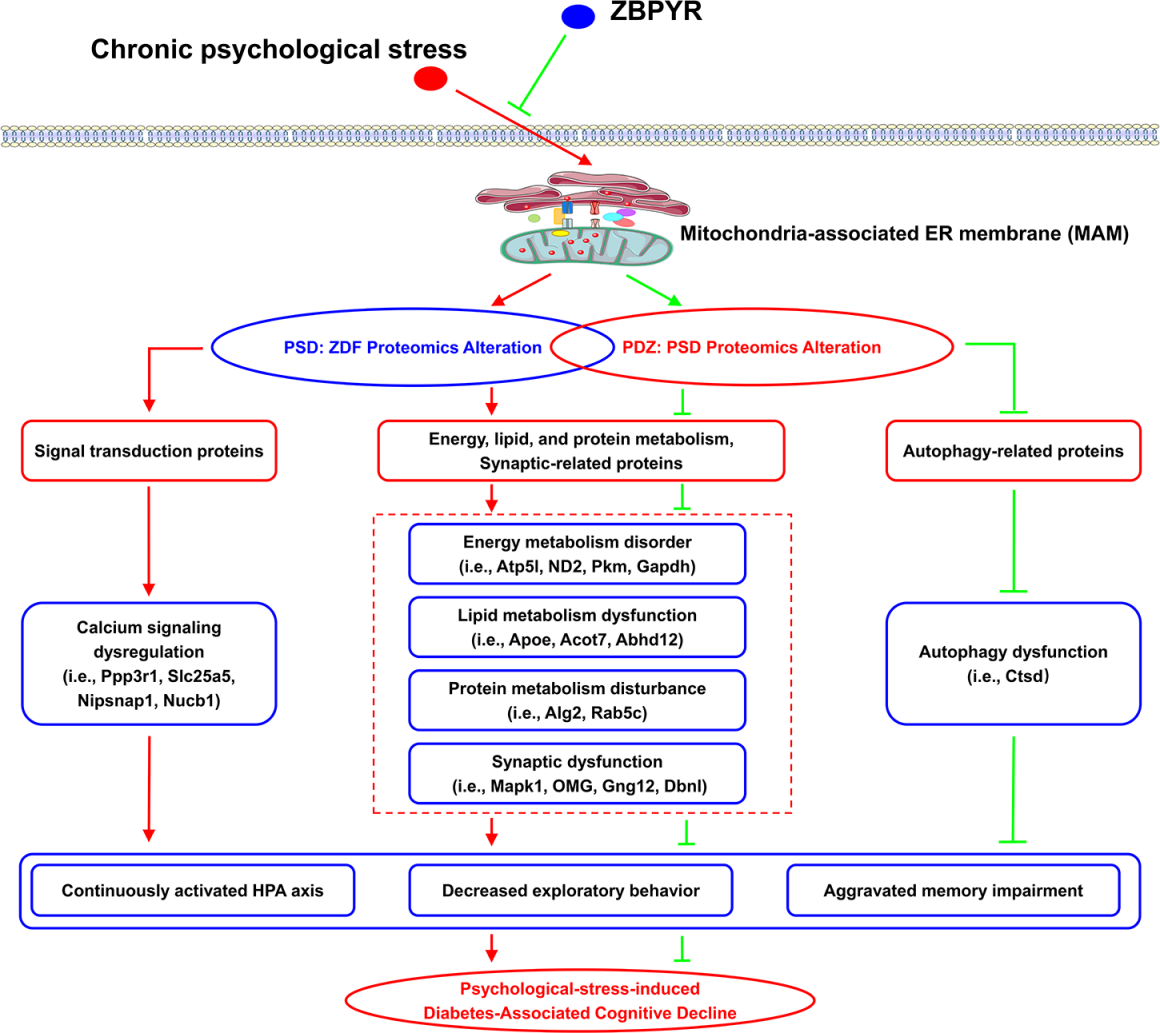
**The potential mechanism of chronic PS and ZBPYR treatment.** Chronic PS causes proteomic alterations in brain MAM of ZDF rats, including in proteins related to lipid and protein metabolism, energy metabolism, synaptic function, and calcium signaling. ZBPYR treatment results in proteomic alterations in PSD rats, including in proteins related to lipid and protein metabolism, energy metabolism, synaptic function, and autophagy. This may trigger the molecular basis of chronic PS aggravated diabetes-associated cognitive decline (i.e., persistent hyperactivity of the HPA axis, decreased exploratory behavior, and aggravated memory impairment) and provides a potential therapeutic mechanism of ZPBYR.

In summary, we demonstrated that chronic PS (restriction, rotation, and congestion) of ZDF rats perpetually interfered with the HPA axis, affected exploratory behaviors, and promoted cognitive anomalies, while ZBPYR treatment significantly improved the above interference. Additionally, elevations in stress-related indicators (i.e. CORT and ACTH) may facilitate the subsequent onset of hyperglycemia in ZDF rats. Proteomic analysis demonstrated that chronic PS is related to changes in the MAM proteome of ZDF rats, including proteins related to OXPHOS (Atp5l and ND2), glycolysis (GAPDH and PKM), lipid metabolism (Apoe, Acot7, and Abhd12), protein metabolism (Alg2 and Rab5c), and synaptic dysfunction (Mapk1, OMG, Gng12, and Dbnl). These proteins were also regulated by ZBPYR administration. Furthermore, calcium signaling and autophagy may play roles in the pathogenesis of PSD and the mechanisms of ZBPYR, respectively. Chronic PS as a common factor in modern life could therefore plausibly contribute to the evolution and progression of T2DM and DACD (Figure 11), while ZBPYR administration may significantly delay and alleviate the occurrence and progression of T2DM and DACD.

## MATERIALS AND METHODS

### Animals and treatment protocol

Male, 6-week-old, obese Zuker diabetic fatty (ZDF) rats were purchased from Vital River Laboratories (Beijing, China) and housed in a specific, pathogen-free animal experimental center in Nanjing University of Chinese Medicine. The animals were fed autoclaved Purina 5008 chow and water *ad libitum* and were housed at 24° C ± 2° C with 65% ± 5% humidity with a 12-hour light-dark cycle. All animal experiments were conducted in accordance with the National Institutes of Health Guide for the Care and Use of Laboratory Animals and were approved by the Animal Ethics Committee of Nanjing University of Chinese Medicine (Approval No. ACU170606).

After one week of acclimatization, the ZDF rats were randomly distributed into three groups (n=8 each): a ZDF control group, a PSD group, and a PDZ group. The PSD and PDZ groups were exposed to three stressful stimulations: restriction, rotation, and congestion all lasting for six weeks. The duration of the restricting stress experiment lasted for 1 hour in weeks 1-2, 1.5 hours in weeks 3-4, and 2 hours in weeks 5-6. The rotation and congestion procedures were performed as previously described in detail [50].

### Preparation and administration of ZBPYR

ZBPYR, consisting of 12 crude herbs, was purchased from Nantong Sanyue Chinese Traditional Medicine Co., Ltd. (Nantong, China) and its preparation and administration was identical to that described in a previous study [50]. During weeks 8 to 15, ZBPYR was administered by oral gavage at a dose of 0.1 mL/10 g body weight to the PDZ group, while the PSD and control groups were administered with an equal dose of ultrapure water.

### Random blood glucose test, oral glucose tolerance test, and insulin tolerance test

The RBG levels in blood samples were measured from weeks 7 to 15. Following chronic PS experiments, all rats were fasted for 14 hours (overnight, for the OGTT) and 6 hours (for the ITT). Blood was sampled at 0, 30, 60, 90, and 120 minutes following glucose (2 g/kg body weight) administration and at 0, 15, 30, 60, 90, and 120 minutes following insulin (0.5 U/kg body weight; Wanbang Biopharmaceuticals Co., Ltd., Xuzhou, China) injection. Blood glucose levels were all determined from tail blood.

### Plasma ACTH and corticosterone assays

Tail blood samples were collected weekly. The samples were centrifuged at 1300×*g* for 10 minutes, and the supernatants were kept frozen at −80° C. Plasma concentrations of ACTH and CORT were measured using a Rat ACTH ELISA kit (Nanjing Jiancheng Bioengineering Institute, Nanjing, China) and a rat CORT ELISA kit (Nanjing Jiancheng Bioengineering Institute). The procedures were conducted in accordance with the instruction manuals, and the data were expressed as ng/ml.

### Behavioral experiments

The OFT was performed during the first day and the MWM was performed during days 2-7 of the last seven days. All procedures were conducted as previously described in detail [50]. The spontaneous locomotor activities were recorded for the OFT. The MWM test consisted of 5-day training for the hidden platform test and the probe trial while the visible platform training was conducted on day seven.

### Isolation of MAM from rat brains

The MAM was isolated according to an established protocol [10] with minor modifications. Briefly, tissues were manually homogenized on ice and nuclei and unbroken cells were pelleted by centrifugation. The supernatant was collected and centrifuged to separate the crude mitochondria from plasma membranes, lysosomes, microsomes, and ER fractions. After several washes, the crude mitochondrial fraction was suspended in 4 ml resuspending buffer III layered on top of 16 ml of a 30% Percoll medium and centrifuged at 95,000×*g* for 30 minutes. The MAM fraction and the pure mitochondrial fraction were separately extracted from the Percoll gradient and were further purified by centrifugation, to remove contaminants. All fractions were flash frozen by liquid nitrogen and preserved at −80° C until use.

### Quantitative proteomic analysis

The iTRAQ quantitative proteomic analysis was performed on triplicate samples of rat brain MAMs and on three independent rat brain MAM protein extractions per group, as described in the Supplementary Materials. After acquiring MS/MS data with a Q-Exactive (Thermo Finnigan, San Jose, CA, USA) mass spectrometer, all data files were processed using the Mascot 2.2 and Proteome Discoverer 1.4 (Thermo) to identify the peptides. The search parameters are detailed in the Supplementary Materials. All reported data were based on 99% confidence for peptide identification, as determined by a false discovery rate (FDR) of no more than 1%. Protein identification was supported by at least one unique peptide identification. Proteins with relative quantification p-values <0.05 and fold changes >1.2 were considered as significant.

### Bioinformatics analysis

The DAVID bioinformatics resource (v6.8) (https://david.ncifcrf.gov/) was used for the GO annotation and KEGG pathway enrichment analysis. STRING (Search Tool for the Retrieval of Interacting Genes/Proteins) database version 11.0 (https://string-db.org/) was used to search for the protein-protein interaction (PPI) networks of the differently regulated proteins. The STRING-generated network was visualized and edited in Cytoscape version 3.7.1. Additionally, R (v3.5.2) was used to generate a Venn diagram and volcano plots to perform the logistic analysis on the MAM proteome.

### Western blot analysis

Samples were lysed in a radio immunoprecipitation assay (RIPA) buffer (Beyotime, China) with a protease and phosphatase inhibitor cocktail (Cell Signaling Technology, USA). The protein samples, measured by a BCA protein assay kit (Beyotime, China), were loaded and resolved by SDS-PAGE, followed by electro-blotting onto the PVDF membranes. The membranes were probed with primary antibodies listed in Supplementary Table 5 and the corresponding HRP-conjugated secondary antibodies. The membranes were developed with an ECL kit (Tanon, China) using an Amersham Imager 600 (General Electric Company, USA).

### Statistical analysis

The data are expressed as means ± standard error (SEM) and analyzed using GraphPad Prism 6.0 statistical software (GraphPad Software, USA). Statistical analyses were performed using Student’s *t* tests for comparing two groups and one-way ANOVAs with Tukey's *post hoc* test for the comparison of three groups. Learning curves were analyzed by two-way repeated-measure ANOVAs and Bonferroni's multiple comparisons *post hoc* tests. The difference was considered to be statistically significant when *p* ≤0.05.

## Supplementary Material

Supplementary Materials

Supplementary Tables
