# Supplementary material for: The ZiBuPiYin recipe regulates proteomic alterations in brain mitochondria-associated ER membranes caused by chronic psychological stress exposure: Implications for cognitive decline in Zucker diabetic fatty rats
Source: Aging (Albany NY). 2020 Nov 18;12(23):23698–726. doi: 10.18632/aging.103894 (PMC7762487; doi:10.18632/aging.103894)
Supplement: Supplementary Materials [file aging-12-103894-s001.pdf]

QUANTITATIVE PROTEOMIC ANALYSIS

Digestion of MAM samples and iTRAQ labeling

100 µl of each sample was digested in parallel via filter-aided sample preparation (FASP). The critical steps of the FASP method are: 200 µl UA buffer (8 M urea and 150 mM Tris-HCl pH 8.0) were added to each sample. DTT was added to the mixed sample to a final concentration of 100 mM and allowed to stand at room temperature for 1.5 h. Then the sample mixture was transferred to an ultrafiltration filter (30 kDa cutoff, Sartorius, Germany) and centrifuged at 13,000×*g* for 20 min, then washed again with UA buffer. Subsequently, 100 µl iodoacetamide solution (50 mM iodoacetamide in UA buffer) was added to the filter. The filter unit was mixed for 1 min followed by incubation for 30 min at room temperature in the dark and centrifuged at 13,000×*g* for 20 min. Two wash steps with 100 µl UA buffer were performed with centrifugation at 13,000×*g* for 20 min after each wash step. Then, 100 µl NH_4HCO_3 buffer (Sigma, St. Louis., MO) was added to the filter and centrifuged at 13,000×*g* for 15 min; this step was repeated thrice. Finally, 40 µl of trypsin (Promega, Madison, WI) buffer (3 µg trypsin in 40 µl NH_4HCO_3 buffer) was added and digested at 37°C for 16–18 h. The filter unit was transferred to a new tube and centrifuged at 13,000×*g* for 30 min. The resulting peptides were collected as a filtrate and desalted with a C18-SD Extraction Disk Cartridge (66872-U Sigma). The peptide concentration was analyzed by OD_{280} .

Subsequently, 50 µg of peptides per sample were labeled with iTRAQ reagents according to the manufacturer's instructions (iTRAQ Reagent-8plex Multiplex Kit, Applied Biosystems SCIEX, Foster City, CA). The MAM samples from ZDF were labeled with reagent 116, the MAM samples from PSD were labeled with reagent 114, the MAM samples from PDZ were labeled with reagent 115, and the IS were labeled with reagent 117. The labeling solution reaction was incubated at room temperature for 1 h prior to further analysis. Then, three independent biological experiments were performed for triplicate LC-MS/MS analyses.

EASY-nLC1000 separation

The column was equilibrated for 20 min with 95% (v/v) solvent A (0.1% (v/v) formic acid in Milli-Q water). Peptide mixtures were first flushed into a sample column, the Thermo Scientific EASY column (2 cm×100 µm, 5 µm-C18), then separated with an analytical column, the Thermo Scientific EASY column (75 µm×100 mm, 3 µm-C18), at 250 nl/min with solvent B (acetonitrile with 0.1% (v/v) formic acid, acetonitrile 84%) using a segmented gradient from 0-55% (v/v) for 220 min, from 55-100% (v/v) for 8 min, and then at 100% (v/v) for 12 min.

MS/MS analysis and quantification

The Q-Exactive (Thermo Finnigan, San Jose, CA) mass spectrometer was set to perform data acquisition in positive ion mode with a selected mass range of 350-1800 mass/charge (*m/z*). The resolving power for the Q-Exactive was set as 70,000 for the MS scan and 17,500 for the MS/MS scan at *m/z* 200. MS/MS data were acquired using the top 10 most abundant precursor ions with charge ≥2 as determined from the MS scan. These were selected with an isolation window of 2 *m/z* and fragmented by higher energy collisional dissociation with normalized collision energies of 29 eV. The maximum ion injection times for the survey scan and the MS/MS scans were 20 and 60 ms, respectively, and the automatic gain control target values for the MS scan mode was set to 3e6. Dynamic exclusion for selected precursor ions was set at 30 s. The underfill ratio was defined as 0.1% on the Q-Exactive.

Raw files were processed using Mascot 2.2 and Proteome Discoverer 1.4 (Thermo). The raw files were searched using the MASCOT engine (Matrix Science, London, UK; v2.2) embedded into Proteome Discoverer 1.4, against the Uniprot Rat database (02-28-2015, 34164 entries). The following search parameters were set: monoisotopic mass values, fragment mass tolerance at 0.1 Da and peptide mass tolerance ± 20 ppm, trypsin as the enzyme, and allowing up to 2 missed cleavages. Fixed modifications were defined as iTRAQ labeling and carbamidomethylation of cysteine; oxidation of methionine was specified as a variable modification. The decoy database pattern was set as the reversed version of the target database. All reported data were based on 99% confidence for peptide identification as determined by a false discovery rate (FDR) of no more than 1%. Protein identification was supported by at least one unique peptide identification.

The iTRAQ analysis of relative protein quantification levels across multiple samples was as follows. Proteome Discoverer 1.4 was used to calculate relative ratios of identified peptides among labeled samples using relative peak intensities of released iTRAQ reporter ions in each of the MS/MS spectra, while relative protein quantification among samples was based on weighted ratios of uniquely identified peptides that

belonged to the specific individual protein in which sample IS was used as a reference. Final ratios of protein quantification were then normalized by the median average protein quantification ratio for unequally mixed differently labeled samples. This correction is based on the assumption that the expression of most proteins does not change. Thus, if samples from each experimental condition are not combined in exactly equal amounts, this normalization fixes the systematic error. Only protein identification that was inferred from the unique peptide identification in all three independent experiments was considered.
