# Supplementary material for: The ZiBuPiYin recipe regulates proteomic alterations in brain mitochondria-associated ER membranes caused by chronic psychological stress exposure: Implications for cognitive decline in Zucker diabetic fatty rats
Source: Aging (Albany NY). 2020 Nov 18;12(23):23698–726. doi: 10.18632/aging.103894 (PMC7762487; doi:10.18632/aging.103894)
Supplement: Supplementary Tables [file aging-12-103894-s002.pdf]

SUPPLEMENTARY TABLES

Supplementary Table 1. Identified proteins known to localize in MAM and associated with diabetes mellitus and cognitive impairment-related disease (UP: unique peptides).

Gene	Diabetes mellitus associated proteins	UP	Note
Nefl	Neurofilament light polypeptide	20	a
Hspd1	60 kDa heat shock protein, mitochondrial	17	a, b
Dld	Dihydrolipoyl dehydrogenase, mitochondrial	13	a, b
Mog	Myelin-oligodendrocyte glycoprotein	10	a
Syn2	Synapsin II, isoform CRA_a	10	a
Map6	Microtubule-associated protein 6	10	b
Gsn	Gelsolin	9	a
Sv2a	Synaptic vesicle glycoprotein 2A	9	a
Psap	Prosaposin	9	a
Ctsd	Cathepsin D	7	a, b
Lrp1	LDL receptor related protein 1	7	a
Marcks	Myristoylated alanine-rich C-kinase substrate	7	a
Nefh	Neurofilament heavy polypeptide	7	a
COX2	cytochrome c oxidase subunit II	6	a, b
Apoe	Apolipoprotein E	5	a, b, c
Pdk1	[Pyruvate dehydrogenase (acetyl-transferring)] kinase isozyme 1, mitochondrial	5	a
S100b	Protein S100-B	4	a, b, c
Eef1a2	Elongation factor 1-alpha 2	4	a, b
Pip4k2b	Phosphatidylinositol-5-phosphate 4-kinase type 2 beta	4	a
Mapk1	Mitogen-activated protein kinase 1	3	a, b
ALG2	ALG2, alpha-1,3/1,6-mannosyltransferase	3	a
Cdc42	Cell division control protein 42 homolog	3	a
Cisd2	CDGSH iron sulfur domain 2	3	a, b, c
Dctn1	Dynactin subunit 1	3	a
Fis1	Mitochondrial fission 1 protein	2	a, c
Tmed10	Transmembrane emp24 domain-containing protein 10	2	a
Dnajc5	DnaJ homolog subfamily C member 5	2	a
Vti1b	Vesicle transport through interaction with t-SNAREs homolog 1B	2	a
Gapdh	Glyceraldehyde-3-phosphate dehydrogenase	1	a, b
ND2	NADH-ubiquinone oxidoreductase chain 2	1	a
Lamp2	Lysosome-associated membrane glycoprotein 2	1	a
Psmd2	26S proteasome non-ATPase regulatory subunit 2	1	a
Cnnm1	Cyclin M1 (Predicted)	1	a
Dbnl	Drebrin-like protein	1	b
Bcap31	B-cell receptor-associated protein 31	1	c

a, Cognitive impairment-related disease associated proteins; b, Diabetes mellitus-associated proteins; c, Known MAM-localized proteins.

Supplementary Table 2. Functional clusters of significant MAM protein changes in PSD:ZDF group.

Sequence name	Gene name	Protein name	UP	FC	p-value
Q6AY58	Bcap31	B-cell receptor-associated protein 31	1	2.68	0.0203
F1LLX8	Lamp2	Lysosome-associated membrane glycoprotein 2	1	2.25	0.0498
Q6IFW6	Krt10	Keratin, type I cytoskeletal 10	6	2.18	0.0367
P30009	Marcks	Myristoylated alanine-rich C-kinase substrate	7	1.48	0.0027
Q68FQ0	Cct5	T-complex protein 1 subunit epsilon	9	1.44	0.0258
Q4FZT9	Psmd2	26S proteasome non-ATPase regulatory subunit 2	1	1.42	0.0130
Q7TNM3	OMG	Oligodendrocyte-myelin glycoprotein	7	1.42	0.0127
P13638	Atp1b2	Sodium/potassium-transporting ATPase subunit beta-2	5	1.39	0.0300
Q9Z270	Vapa	Vesicle-associated membrane protein-associated protein A	6	1.38	0.0276
Q6JAM9	Tmem35	Transmembrane protein 35	1	1.38	0.0094
D3ZEI4	Hepacam	Hepatocyte cell adhesion molecule	3	1.37	0.0308
P31647	Slc6a11	Sodium- and chloride-dependent GABA transporter 3	8	1.37	0.0464
P02650	Apoe	Apolipoprotein E	5	1.34	0.0421
B0BNM7	Sco1	Sco1 protein	1	1.34	0.0130
Q6AY84	Scrn1	Secernin-1	5	1.32	0.0062
O88377	Pip4k2b	Phosphatidylinositol 5-phosphate 4-kinase type-2 beta	4	1.32	0.0283
P23562	Slc4a1	Band 3 anion transport protein	8	1.32	0.0086
P63086	Mapk1	Mitogen-activated protein kinase 1	3	1.32	0.0192
Q63345	Mog	Myelin-oligodendrocyte glycoprotein	10	1.32	0.0342
Q812E9	Gpm6a	Neuronal membrane glycoprotein M6-a	5	1.29	0.0495
Q6PST4	Atl1	Atlastin-1	3	1.28	0.0175
D4AAE9	Cisd2	CDGSH iron sulfur domain 2	3	1.28	0.0167
G3V7P1	Stx12	Syntaxin-12	3	1.27	0.0016
Q3MHS9	Cct6a	Chaperonin containing Tcp1, subunit 6A (Zeta 1)	7	1.27	0.0099
Q5RKJ9	RAB10	RAB10, member RAS oncogene family	6	1.26	0.0105
F1LMR7	Dpp6	Dipeptidyl aminopeptidase-like protein 6	9	1.26	0.0169
Q68FP1	Gsn	Gelsolin	9	1.26	0.0487
G3V9B3	Mag	Myelin-associated glycoprotein	10	1.25	0.0017
Q02563	Sv2a	Synaptic vesicle glycoprotein 2A	9	1.24	0.0444
Q5RKI0	Wdr1	WD repeat-containing protein 1	8	1.24	0.0372
A0A096MJM1	Rhog	Ras homolog family member G	7	1.24	0.0019
P62909	Rps3	40S ribosomal protein S3	3	1.23	0.0125
P63322	Rala	Ras-related protein Ral-A	4	1.22	0.0347
Q6PEC4	Skp1	S-phase kinase-associated protein 1	3	1.22	0.0027
Q5FVQ4	Mlec	Malectin	3	1.22	0.0167
P97710	Sirpa	Tyrosine-protein phosphatase non-receptor type substrate 1	6	1.21	0.0176
B0BNM1	Apoa1bp	NAD(P)H-hydrate epimerase	3	0.83	0.0340
P63100	Ppp3r1	Calcineurin subunit B type 1	6	0.83	0.0433
G3V6P8	Gng12	Guanine nucleotide-binding protein subunit gamma	3	0.82	0.0103
P23965	Eci1	Enoyl-CoA delta isomerase 1, mitochondrial	7	0.82	0.0254
G3V733	Syn2	Synapsin II, isoform CRA_a	10	0.82	0.0197
Q5XIH3	Ndufv1	NADH dehydrogenase [ubiquinone] flavoprotein 1, mitochondrial	16	0.81	0.0425
P38718	Mpc2	Mitochondrial pyruvate carrier 2	3	0.81	0.0186
Q09073	Slc25a5	ADP/ATP translocase 2	2	0.81	0.0123
Q5EBA4	Nipsnap1	Nipsnap1 protein	7	0.79	0.0426
F1M6X5	Txnrd2	Thioredoxin reductase 2, mitochondrial	6	0.78	0.0449
P19627	Gnaz	Guanine nucleotide-binding protein G(z) subunit alpha	5	0.78	0.0298
P41565	Idh3g	Isocitrate dehydrogenase [NAD] subunit gamma 1, mitochondrial	8	0.78	0.0090
P60905	Dnajc5	DnaJ homolog subfamily C member 5	2	0.77	0.0353

D4A1C0	Cnnm1	Cyclin M1 (Predicted)	1	0.76	0.0407
B2GV06	Oxct1	Succinyl-CoA:3-ketoacid coenzyme A transferase 1, mitochondrial	17	0.76	0.0343
G3V6H5	Slc25a11	Mitochondrial 2-oxoglutarate/malate carrier protein	13	0.75	0.0444
Q80W89	Ndufa11	NADH dehydrogenase [ubiquinone] 1 alpha subcomplex subunit 11	3	0.74	0.0145
F1MA54	Pdk1	[Pyruvate dehydrogenase (acetyl-transferring)] kinase isozyme 1, mitochondrial	5	0.74	0.0411
F1LRZ7	Nefh	Neurofilament heavy polypeptide	7	0.72	0.0150
Q6TUG0	Dnajb11	DnaJ homolog subfamily B member 11	1	0.72	0.0287
F8WG67	Acot7	Acyl-CoA thioesterase 7, isoform CRA_a	3	0.71	0.0040
B4F7C2	Tubb4a	Tubulin beta chain	5	0.70	0.0201
B2GV73	Arpc3	Actin-related protein 2/3 complex subunit 3	1	0.69	0.0095
A0A097PE04	COX2	Cytochrome c oxidase subunit 2	6	0.69	0.0311
Q6P6R2	Dld	Dihydrolipoyl dehydrogenase, mitochondrial	13	0.68	0.0303
Q8CFN2	Cdc42	Cell division control protein 42 homolog	3	0.68	0.0243
P56522	Fdxr	NADPH:adrenodoxin oxidoreductase, mitochondrial	9	0.68	0.0084
Q9R170	Bcat1	Branched-chain-amino-acid aminotransferase	3	0.68	0.0341
P49432	Pdhb	Pyruvate dehydrogenase E1 component subunit beta, mitochondrial	13	0.66	0.0235
P63039	Hspd1	60 kDa heat shock protein, mitochondrial	17	0.65	0.0438
Q6PDU7	Atp5l	ATP synthase subunit g, mitochondrial	3	0.64	0.0007
P19527	Nefl	Neurofilament light polypeptide	20	0.64	0.0170
P04631	S100b	Protein S100-B	4	0.64	0.0053
B0BNK1	Rab5c	RAB5C, member RAS oncogene family	4	0.63	0.0087
Q5I0L3	Yars2	Tyrosine--tRNA ligase, mitochondrial	2	0.63	0.0157
Q9JHL4	Dbnl	Drebrin-like protein	1	0.59	0.0112
Q6P7S0	Pkm	Pyruvate kinase	2	0.58	0.0106
P28023	Dctn1	Dynactin subunit 1	3	0.58	0.0256
Q6AYT7	Abhd12	Monoacylglycerol lipase ABHD12	1	0.56	0.0410
Q63083	Nucb1	Nucleobindin-1	2	0.56	0.0479
Q5XIG4	Ociad1	OCIA domain-containing protein 1	1	0.54	0.0040
P84817	Fis1	Mitochondrial fission 1 protein	2	0.53	0.0175
D4A7V1	Sh3glb2	Endophilin-B2	4	0.50	0.0353
P04797	Gapdh	Glyceraldehyde-3-phosphate dehydrogenase	1	0.46	0.0095
Q06QE9	ND2	NADH-ubiquinone oxidoreductase chain 2	1	0.42	0.0158
F1LNC4	Vti1b	Vesicle transport through interaction with t-SNAREs homolog 1B	2	0.40	0.0218
Q6Q0N1	Cndp2	Cytosolic non-specific dipeptidase	3	0.33	0.0048
Q45QL2	Gnb4	Guanine nucleotide binding protein beta-4	2	0.33	0.0416
G3V6U3	Alg2	ALG2, alpha-1,3/1,6-mannosyltransferase	3	0.31	0.0033

The color gradient of green and yellow were used to visualize the increased or decreased abundances of PSD proteins compared to the ZDF group, respectively (FC: fold change).

Supplementary Table 3. Functional clusters of significant MAM protein changes in PDZ:PSD group.

Sequence name	Gene name	Protein name	UP	FC	p-value
G3V6U3	Alg2	ALG2, alpha-1,3/1,6-mannosyltransferase	3	2.87	0.0070
Q6Q0N1	Cndp2	Cytosolic non-specific dipeptidase	3	2.27	0.0088
Q6AYT7	Abhd12	Monoacylglycerol lipase ABHD12	1	2.07	0.0453
W0NT55	Slc4a10	Anion exchange protein	3	1.82	0.0214
P04631	S100b	Protein S100-B	4	1.73	0.0095
P84817	Fis1	Mitochondrial fission 1 protein	2	1.70	0.0418
P04797	Gapdh	Glyceraldehyde-3-phosphate dehydrogenase	1	1.67	0.0473
Q06QE9	ND2	NADH-ubiquinone oxidoreductase chain 2	1	1.66	0.0315
Q3T1K5	Capza2	F-actin-capping protein subunit alpha-2	2	1.63	0.0391
Q6P7S0	Pkm	Pyruvate kinase	2	1.51	0.0267
D3ZZ21	Ndufb6	NADH dehydrogenase (Ubiquinone) 1 beta subcomplex, 6 (Predicted)	1	1.47	0.0273
Q6P7A4	Psap	Prosaposin	9	1.45	0.0496
Q9JHL4	Dbnl	Drebrin-like protein	1	1.44	0.0461
Q6PDU7	Atp5l	ATP synthase subunit g, mitochondrial	3	1.42	0.0414
B0BNK1	Rab5c	RAB5C, member RAS oncogene family	4	1.41	0.0138
Q6TUG0	Dnajb11	DnaJ homolog subfamily B member 11	1	1.37	0.0423
P24268	Ctsd	Cathepsin D	7	1.36	0.0141
Q63584	Tmed10	Transmembrane emp24 domain-containing protein 10	2	1.36	0.0089
P56522	Fdxr	NADPH:adrenodoxin oxidoreductase, mitochondrial	9	1.33	0.0155
F1M6X5	Txnrd2	Thioredoxin reductase 2, mitochondrial	6	1.32	0.0462
Q9R170	Bcat1	Branched-chain-amino-acid aminotransferase, cytosolic	3	1.28	0.0449
F8WG67	Acot7	Acyl-CoA thioesterase 7, isoform CRA_a	3	1.28	0.0352
Q62703	Rcn2	Reticulocalbin-2	4	1.23	0.0254
G3V6P8	Gng12	Guanine nucleotide-binding protein subunit gamma	3	1.23	0.0009
G3V928	Lrp1	LDL receptor-related protein 1	7	1.21	0.0329
Q63560	Map6	Microtubule-associated protein 6	10	0.83	0.0393
P02650	Apoe	Apolipoprotein E	5	0.83	0.0359
G3V9B3	Mag	Myelin-associated glycoprotein	10	0.83	0.0041
P62632	Eef1a2	Elongation factor 1-alpha 2	4	0.81	0.0137
Q63327	Mobp	Myelin-associated oligodendrocyte basic protein	3	0.81	0.0261
P63086	Mapk1	Mitogen-activated protein kinase 1	3	0.80	0.0295
Q7TNM3	OMG	Oligodendrocyte-myelin glycoprotein	7	0.77	0.0290
O35987	Nsfl1c	NSFL1 cofactor p47	1	0.66	0.0028

The color gradient of green and yellow were used to visualize the increased or decreased abundances of PDZ proteins compared to the PSD group, respectively.

Supplementary Table 4. Functional clusters of significant MAM protein changes in PSD:ZDF and PDZ:PSD groups.

Sequence name	Gene name	Protein name	UP	PSD: ZDF		PDZ:PSD	
				FC	p-value	FC	p-value
Q7TNM3	OMG	Oligodendrocyte-myelin glycoprotein	7	1.42	0.0127	0.77	0.0290
P02650	Apoe	Apolipoprotein E	5	1.34	0.0421	0.83	0.0359
P63086	Mapk1	Mitogen-activated protein kinase 1	3	1.32	0.0192	0.80	0.0295
G3V9B3	Mag	Myelin-associated glycoprotein	10	1.25	0.0017	0.83	0.0041
G3V6P8	Gng12	Guanine nucleotide-binding protein subunit gamma	3	0.82	0.0103	1.23	0.0009
F1M6X5	Txnrd2	Thioredoxin reductase 2, mitochondrial	6	0.78	0.0449	1.32	0.0462
Q6TUG0	Dnajb11	DnaJ homolog subfamily B member 11	1	0.72	0.0287	1.37	0.0423
F8WG67	Acot7	Acyl-CoA thioesterase 7, isoform CRA_a	3	0.71	0.0040	1.28	0.0352
P56522	Fdxr	NADPH:adrenodoxin oxidoreductase, mitochondrial	9	0.68	0.0084	1.33	0.0155
Q9R170	Bcat1	Branched-chain-amino-acid aminotransferase, cytosolic	3	0.68	0.0341	1.28	0.0449
Q6PDU7	Atp5l	ATP synthase subunit g, mitochondrial	3	0.64	0.0007	1.42	0.0414
P04631	S100b	Protein S100-B	4	0.64	0.0053	1.73	0.0095
B0BNK1	Rab5c	RAB5C, member RAS oncogene family	4	0.63	0.0087	1.41	0.0138
D3ZQQ5	Dnm1	Dynamin-1	4	0.60	0.0133	1.50	0.0445
Q9JHL4	Dbnl	Drebrin-like protein	1	0.59	0.0112	1.44	0.0461
Q6P7S0	Pkm	Pyruvate kinase	2	0.58	0.0106	1.51	0.0267
Q6AYT7	Abhd12	Monoacylglycerol lipase ABHD12	1	0.56	0.0410	2.07	0.0453
P84817	Fis1	Mitochondrial fission 1 protein	2	0.53	0.0175	1.70	0.0418
P04797	Gapdh	Glyceraldehyde-3-phosphate dehydrogenase	1	0.46	0.0095	1.67	0.0473
Q06QE9	ND2	NADH-ubiquinone oxidoreductase chain 2	1	0.42	0.0158	1.66	0.0315
Q6Q0N1	Cndp2	Cytosolic non-specific dipeptidase	3	0.33	0.0048	2.27	0.0088
G3V6U3	Alg2	ALG2, alpha-1,3/1,6-mannosyltransferase	3	0.31	0.0033	2.87	0.0070

The color gradient of green and yellow were used to visualize the increased or decreased abundances, respectively.

Supplementary Table 5. Antibodies applied in Western blotting (WB).

Antibody	WB Dilution	Catalog No.	Provider
anti-FACL4	1:1000	ab155282	Abcam
anti-cytochrome c	1:500	4280	Cell Signaling Technology
anti-alpha tubulin	1: 1000	ab176560	Abcam
anti-KDEL	1:1000	ADI-SPA-827-D	Enzo Life Sciences
anti-IP3 receptor 1	1:1000	8568	Cell Signaling Technology
anti-VDAC	1:1000	4866	Cell Signaling Technology
anti-PKM	1:500	ab38237	Abcam
anti ERK2	1:1000	ab32081	Abcam
anti-apolipoprotein E	1:500	ab183597	Abcam
anti-GAPDH	1:1000	5174	Cell Signaling Technology
anti-ALG2	1:1000	ab183597	Abcam
